# Applications of flow cytometry in equine medicine: current clinical and research perspectives

**DOI:** 10.3389/fvets.2026.1954604

**Published:** 2026-10-15

**Authors:** Agnieszka Żak-Bochenek, Daria Lorek, Agnieszka Partyka, Natalia Siwińska

**Affiliations:** 1 Department of Immunology, Pathophysiology and Veterinary Preventive Medicine, Wrocław University of Environmental and Life Sciences, Wrocław, Poland; 2 Department of Reproduction and Clinic of Farm Animals, Wrocław University of Environmental and Life Sciences, Wrocław, Poland; 3 Department of Internal Medicine with Horse, Dog and Cat Clinic, Wrocław University of Environmental and Life Sciences, Wrocław, Poland

**Keywords:** flow cytometry, horses, immunodeficiency, immunophenotyping, leukemia, lymphoma, semen evaluation

## Abstract

Flow cytometry is a powerful analytical technique that enables rapid, quantitative, and multiparametric assessment of individual cells in suspension. Owing to its ability to evaluate cellular morphology, viability, and the expression of specific surface and intracellular markers, it has become an indispensable tool in biomedical research and clinical diagnostics. Over the past two decades, significant advances in cytometric technologies and the increasing availability of species-specific reagents have expanded the applicability of flow cytometry in equine medicine. This review highlights the predominantly research-based applications of flow cytometry in horses and their potential to address diagnostic gaps and complement conventional diagnostic methods. Particular attention is given to its principal applications in equine clinical immunology, including peripheral blood lymphocyte immunophenotyping; oncology, through the characterization of hematopoietic and lymphoid neoplasms; internal medicine, via cytometric evaluation of bronchoalveolar lavage fluid; and andrology, where flow cytometry serves as a valuable tool for the assessment of stallion semen quality. The article also highlights recent developments and discusses the growing role of flow cytometry as a diagnostic and research platform in contemporary equine medicine.

## 1 Introduction

Flow cytometry is an advanced, quantitative technique for the analysis of individual cells, known since the 1960s. This method enables simultaneous assessment of properties such as cell size and granularity, as well as the evaluation of cell viability using dyes that distinguish live and dead cells based on membrane integrity or metabolic activity. In addition, it allows the detection of specific molecular markers located on the cell surface or intracellularly, through the use of fluorescently labeled antibodies. A flow cytometer typically comprises multiple lasers of distinct excitation wavelengths, an optical detection system equipped with photodetectors, and a hydrodynamic focusing module that aligns cells in single file for interrogation (1). The capacity for multiparametric, quantitative, single-cell analysis underlies the exceptional utility of flow cytometry in the biological sciences, including both human and veterinary medicine. Furthermore, flow cytometry is a rapidly evolving technology, with recent advances encompassing, for example, the development of imaging flow cytometry systems equipped with charge-coupled device (CCD) cameras (1, 2). Flow cytometry enables rapid, multi-parametric evaluation of individual cells in suspension, which permits the analysis of a wide array of cellular biological materials. These may include peripheral blood, bone marrow, bronchoalveolar lavage fluid, fine-needle aspirates from various organs or tumors, semen, body cavity fluids and *in vitro* cultured cell lines (3). A comprehensive overview of all applications of flow cytometry in basic research, applied biological sciences, and human medicine, as well as a detailed discussion of the highly complex aspects of cytometric methodologies, instrumentation, panel design, and sample preparation, is beyond the scope of this article. The objective of the present work is instead to summarize current knowledge on the use of flow cytometry in equine medicine, both in the research setting and in commercial, routine diagnostic practice. The first review dedicated to this topic was published in 2002 by Davis et al. (3); however, during the ensuing two decades, both our understanding of the technique and its clinical implementation in hippiatry have expanded substantially. Flow cytometry has been extensively validated and adapted for routine clinical diagnostics in human medicine, including the use of dedicated diagnostic flow cytometers. In equine medicine, however, flow cytometry remains largely a research-based tool, with diagnostic applications still lacking standardized and validated protocols. Currently, many of the analyses mentioned are being conducted at research centers for scientific purposes; however, they appear to have the potential to fill a diagnostic gap in the future and provide significant support for basic diagnostic methods. In equine samples analysis, the principal clinical applications of flow cytometry include: clinical immunology (immunophenotyping of peripheral blood lymphocytes, PBL), oncology (immunophenotyping of neoplastic cells from peripheral blood, bone marrow, and lymph node aspirates), internal medicine (cytometric evaluation of bronchoalveolar lavage fluid—BALF), and andrology (assessment of stallion semen quality) (3, 4).

## 2 Search strategy

This work is a narrative review, rather than as a full systematic review with meta-analysis. Literature searches were conducted in PubMed, Scopus and MadBarn Equine Research Bank (https://madbarn.com/research/) using combinations of keywords related to the species (“equine”, “horse”), the methodology (“flow cytometry”, “immunophenotyping”, “leukocyte antigens”, “monoclonal antibodies”, “cluster of differentiation”), pathological conditions (“immunodeficiencies”, “leukemia”, “lymphoma”), and the biological specimen or matrix (“peripheral blood mononuclear cells”, “PBMC”, “peripheral blood leukocytes”, “blood”, “bone marrow”, “bronchoalveolar lavage fluid”, “BALF”, “semen”, “sperm”). Data from our own research and cytometric analyses were also incorporated as supplementary sources of images.

## 3 Immunophenotyping

Cellular immunophenotyping relies on the application of direct or indirect immunofluorescence techniques. Antigens characteristic of defined cell subpopulations, designated as clusters of differentiation (CD), are identified using antigen-specific antibodies conjugated to a fluorochrome or, in the case of the indirect approach, by employing an appropriately labeled secondary antibody. For many years, the routine commercial application of flow cytometry in horses was constrained by the limited availability of monoclonal antibodies cross-reactive with equine leukocytes. Since the 1990s, however, an expanded antibody panel has been established, either by selecting reactive clones from existing anti-human monoclonal antibodies or by generating monoclonal antibodies specifically directed against equine antigens. Initial efforts focused on defining antibodies specific for fundamental leukocyte markers, including CD2, CD3, CD4, CD5, CD8, CD11a/18, CD13, CD44, as well as major histocompatibility complex (MHC) class I and class II (5, 6). Subsequently, the panel was broadened to include B- and T-cell–associated markers such as CD19 and CD28 (7) and later further extended by the CD18, CD29, and DR (8). More recently, advances in reagent availability have enabled the phenotypic characterization of equine regulatory T cells, employing the extracellular marker CD25 in combination with the intracellular transcription factor FoxP3 (9, 10). An overview of the antibodies currently available for the detection of equine extracellular antigens, including details on specific clones is presented in Table 1.

**Table 1:** The overview of antibodies that recognize extracellular antigens specific to defined cellular subsets and enable their identification by flow cytometric analysis in horses.

Target antigen	Equine specific clones	Other validated clones	Cells subpopulation	Comment
CD2	MAC288, IFA6, HB88a (5, 6)	39C1.5 (153)	T cells	Equine CD2 is predominantly expressed on a substantial proportion of peripheral blood lymphocytes, encompassing the majority of T lymphocytes and a minor subset of B lymphocytes, while being absent from granulocytes (5, 6). Immunohistochemical analyses demonstrate robust expression on most T lymphocytes within the paracortical regions of lymph nodes, with only occasional positively stained lymphocytes observed within follicular areas (5, 6).
CD3	F6G.3 (G12) (6, 154, 155)		T cells	CD3 is widely recognized as a pan–T lymphocyte marker in the horse and is commonly used to identify the global equine T cell population (5, 6, 155).
CD4	CVS4 (5, 6) HB61A (5)		Th cells	Some manufacturers also offer clones (HB86 and HB65A) recognizing EqWC4 described as CD4-detecting (11).
CD5	E23B, HB80A, HB19A, RVC1, HT23A (5) CVS5 (5, 6) F13C.3(D9), TH2A10 (6)		Lymphocytes	Equine CD5 is predominantly expressed on the majority of T lymphocytes; however, it has also been reported that equine CD5 can be detected at very low levels on B lymphocytes and granulocytes (156).
CD8	CVS8 (5, 6) HT14A, (5) CVS21, F18H.2 (6), 73/6.9.1 (157)		Tc cells	It should be noted that the CVS21 clone also partially recognizes equine CD3^−^ lymphocytes, which exhibit CD21 and surface IgG expression. Therefore, when employing the CVS21 clone for cell labeling, it is advisable to use it in conjunction with CD3^+^ staining to ensure accurate discrimination of T cells from CD3^−^ populations (155). CD8 is a transmembrane glycoprotein that serves as a co-receptor for the T cell receptor (TCR). There are two isoforms of the protein, CD8 alpha and CD8 beta, recognized by different clones (73/6.9.1 and HT14A, respectively).
CD11a/18	CVS9, H20A, HB20A (5) CZ3.2 (5, 6)			Identifies the equine homolog of the human CD11a/CD18 cell surface antigen, a heterodimeric integrin complex expressed on all equine cells of hematopoietic origin (5). The expression of equine CD11a/CD18 is up regulated in specific T-lymphocyte subpopulations.
CD11c	1C4 (158)			Population: CD11c^+^MHC-II^+^TLR4^+^CD14^low^ could be considered as putative dendritic cells (158).
CD13	CVS19 (5, 6)			CD13 is broadly expressed across multiple cell types of myeloid origin, including all circulating neutrophils, basophils, monocytes, as well as fibroblasts, renal epithelial cells, endothelial cells, and mesenchymal stem cells. In contrast, it is not expressed by cells of the lymphoid lineage, such as T and B cells (5, 6). May be used for differentiating myeloid and lymphoid lineage cells in tumors of the haematopoietic system.
CD14	105 (57, 159) CZ2.2, (5, 6, 160)		Monocytes/macrophages	CD14 is a glycosylphosphatidylinositol (GPI)-anchored membrane glycoprotein that functions as a monocyte/macrophage differentiation antigen. By employing an anti-CD14 antibody in combination with an anti-IgE antibody, it is possible to isolate the CD14^−^IgE^+^ cell population, which comprises basophils and mast cells. (57).
CD16	9G5 (161)		FcγRIII	Subpopulation of monocytes CD14^+^ CD16^+^ (9, 161).
CD19-like	CZ2.1 (5, 6, 156)		B cells	The currently available repertoire of equine B-cell–specific antibodies lacks a broadly reactive, lineage-defining surface marker analogous to human or murine CD19 or CD79a, which are widely used in immunological studies. A monoclonal antibody recognizing the CZ2.1 epitope has been reported to label equine B lymphocytes and is often described as a “CD19-like” marker; however, the precise molecular identity of its target antigen remains undetermined (7). The Pan-B cell antibody clone CVS36 has been shown to recognize all immunoglobulin isotypes and is reported to stain 100% of CD5-negative peripheral blood mononuclear cells (PBMCs) (6). For general applications in which comprehensive coverage of the B-cell compartment is desired, it may be advantageous to employ a combination of CD8, CD4, and Pan-B antibodies for sorting equine PBMCs, whereas the use of IgG1, IgM, and/or CD21 antibodies in various combinations may be preferable for the isolation or characterization of specific B-cell subpopulations (155). CD79α, also referred to as mb-1, is a transmembrane protein with an approximate molecular mass of 45 kDa that is expressed by B lymphocytes throughout their differentiation, from the early pre–B-cell stage to the plasma cell stage. In flow cytometric or related immunophenotype analyses, both its extracellular and intracellular domains can be detected using appropriate antibody-based staining strategies.
CD21		B-ly4 (156, 162)		
Pan B	CVS36 (155), MAC292 (5)	B29A (5)		
IgM		CM7 (45)		
CD79α		HM57 (7)		
CD23	51-3 (163)		FcεRII	Subpopulations of CD23^+^IgM^+^ and CD23^+^IgG1^+^ B cells (163).
CD25	15-1 (9)	AF223NA (164)	Regulatory T cells	The analysis can be supplemented with the intracellular marker FoxP3 [clones PCH101 (164) or FJK-16s (68, 165)].
CD34		1H6 (45)	Progenitor cells	According to the work of Maia et al. (166), clone 581 (mouse anti-human CD34) does not react with equine MSCs derived from bone marrow.
CD44	CZ5.15, CVS18 (5, 6)	BAT31A (5), H22A (12, 162)	Leukocytes	CD44, a glycosylated transmembrane protein of 65–100 kDa, is widely expressed on the surface of most cell types (162).
CD45		DH16A (12)	Leukocytes	
172a		DH59B (12)	Myeloid cells	Also referred to as SIRPα or MyD-1 antigen, CD172a is a transmembrane signal-regulatory protein predominantly expressed on cells of the myeloid lineage, including macrophages, monocytes, dendritic cells, granulocytes, myeloid progenitors, and hematopoietic stem cells, as well as on neuronal cells (167).
CD154 (CD40L)		IDEc-131 (168)	Subpopulation of activated Th cells	
granulocyte	HACT39A (169)	DH24A (57, 63)	Neutrophils/granulocytes	An anti-dog CD90 antibody (clone DH24A) is available that distinguishes equine neutrophils (57, 160).
MHC class I	CVS22 (5) CZ6, MAC291 (5, 6) CZ3 (45)	H58A (12, 162)	All nucleated cells	The major histocompatibility complex (MHC) is a cluster of genes referred to as the equine leukocyte antigen (ELA) region.
MHC class II	CZ11 (5, 6) CVS20 (6)	H42A (12, 162), TH14B (3)	Antigen presenting cells ^*^	Clone CVS20 reacts with all equine B cells and 95% of equine T cells. ^*^Note! MHC class II expression on equine cells differs significantly from expression in humans and mice, covering 40%−98% of T lymphocytes, including both CD4^+^ and CD8^+^ cells (170).
NCR1 (NKp46)	4F2 (171)		NCR1^+^ subpopulation of NK cells	
IgE		176 (172)	Basophils	Basophils: (IgE^+^MHCII^low^CD14^−^), IgE binding monocytes: (IgE^+^CD14^+^CD16^−^) IgE^+^ plasmablasts (IgE^+^CD23^high^) (57).

The examples encompass both equine-specific monoclonal antibodies and antibodies originally developed for other species but with experimentally verified cross-reactivity in the horse. The application of each antibody clone is substantiated by data reported in the scientific literature.

The panels additionally comprise antibodies directed against EqWC1, a ubiquitous cell surface antigen expressed on equine leukocytes; EqWC2, which is predominantly expressed on T lymphocytes and granulocytes, with lower expression detected on a minor subset of B lymphocytes; and EqWC4, which is restricted to a specific subset of equine T cells and is frequently designated as anti-CD4 by commercial suppliers (clones HB86 and HB65A) (11). Moreover, the panels include mono- and polyclonal antibodies directed against the heavy chains of defined immunoglobulin isotypes (3, 5, 6). Numerous studies have focused on evaluating the cross-reactivity of various human-reacting antibodies with equine antigens, thereby expanding the repertoire of immunological markers available for this species (8, 9, 12). A number of monoclonal antibodies specifically designed for horses have also been developed, as described in detail by Wagner et al. (9) in 2024. Analogous to other species, basic morphometric cytometric analysis of blood cells is based on their differentiation according to size (using a forward scatter detector—FSC) and granularity intensity (based on a side scatter detector—SSC), usually in a linear (rarely logarithmic) system. The dominant populations include lymphocytes, monocytes, and granulocytes, but in the latter group, a separate population of eosinophils and basophils can be distinguished (12). In equine blood samples, compared to other animal species, a high granularity content in eosinophils is characteristic, which causes a clear separation of this cell population from other granulocytes (12, 13).

Numerous studies have examined the influence of physiological variables, such as age and sex, as well as individual and environmental factors (e.g., sampling time, or breed), on the abundance and morphometric properties of distinct leukocyte populations. Research by Schnabel et al. (14), based on the analysis of SSC and FSC leukocytes, demonstrated slight age-related differences, along with minor effects associated with breed and time of sampling. In a cohort of 24 horses aged 2–21 years, an age-dependent decrease in the percentage of lymphocytes and a concomitant increase in the percentages of granulocytes and monocytes were observed, accompanied by an increase in cell size across these populations (14). The authors reported mean relative values for individual white blood cell (WBC) populations of 21.52% lymphocytes, 5.71% monocytes, and 72.89% granulocytes, without differentiation between eosinophils and basophils (14). These values did not vary from established reference intervals for the complete blood count (CBC) in horses. When establishing reference intervals for lymphocyte subpopulations in horses, investigators have primarily focused on characterizing changes occurring from birth until immunological maturation. Across all studies, the total leukocyte and lymphocyte counts were markedly higher in neonatal foals and during the first months of life than in adult horses (15–17). Around 1 month of age a distinct increase in both the absolute number and percentage of CD4^+^ and CD8^+^ T cells is observed, although in the study by Smith et al. (15) the percentage of CD8^+^ cells decreased. Absolute circulating CD4^+^ and CD8^+^ T-lymphocyte concentrations increased linearly up to 3 months of age; however, during the same period, the proportional representation of circulating CD4^+^ cells exhibited a slight decline by 4 months of age. In adult age, horses demonstrate a reduction in total CD4^+^ and CD8^+^ T-cell counts, while maintaining a percentage distribution of these subsets that is comparable to that observed in neonates (17). In most cases, however, no significant differences in the CD4:CD8 ratio were detected. The most pronounced differences in the proportions of lymphocyte subsets between foals and adult horses were observed for B cells. Both the absolute count and the percentage of circulating B cells immediately after birth are markedly higher in foals than in adult horses, and these values continue to increase during the first weeks of life, remaining significantly elevated for the first several months postpartum. According to Frymus and Scholleberger (18), this parameter reaches a stable level at approximately 5 months of age, whereas Flaminio et al. (17) report stabilization much later; these discrepancies may result from the methodologies used for B cells analysis. Satué et al. (19), in a study conducted on a cohort of 159 young Andalusian horses, demonstrated that the proportion of B lymphocytes reaches a stable level at approximately 3 years of age. A research team from Portugal also investigated the influence of the reproductive cycle in Lusitano mares on lymphocyte subpopulations; however, no significant differences were detected among the anestrous, follicular, and luteal phases (20–22). During the postpartum period, the proportions of CD3^+^, CD2^+^, and B lymphocytes were reduced compared with levels observed in the third trimester of pregnancy. In addition, a concurrent decline in the overall numbers of CD4^+^ and CD8^+^ T lymphocytes, as well as in total T cells, was noted during this interval (20). In the study mentioned above, the proportion of B cells was markedly higher than that reported in other publications (approximately 30% of total lymphocytes). This discrepancy may be attributable to the method used for their quantification, specifically the lack of direct labeling with an anti-CD19 antibody (or equivalent) and the consequent necessity to estimate their number indirectly by subtracting the count of CD2^+^ cells from the total lymphocyte count. Until now, reported values for adult horses were established based on analyses of 6 and 10 clinically healthy individuals, respectively, and are summarized in Table 2 (3, 23), however, both the variability among the clones used and the small sample size make it impossible to determine reference intervals. In Figure 1 the author present an example of the cell distribution of PBMC of clinically healthy adult horse. The investigations conducted by McFarlane et al. (24) in geriatric horses (>20 years of age) also demonstrated a significant age-associated decline in the total lymphocyte count, encompassing CD5^+^, CD4^+^, CD8^+^ T cells as well as B cells, in parallel with a reduction in the proportion of CD8^+^ cells. Additionally, a significant increase in the CD4:CD8 ratio was observed in this group, further supporting the concomitant presence of inflammaging (chronic, low-grade systemic inflammation) and immunosenescence (age-associated functional decline and exhaustion of the immune system) in geriatric horses (25).

**Table 2:** Reported values for the relative proportions (%) of distinct lymphocyte subpopulations in clinically healthy adult horses (mean ± SD and/or range), derived from a comprehensive review of the published literature.

References	Pellegrini-Masini et al. (23)		Davis et al. (3)	
*N*	6		10	
T lymphocytes				
Marker	% of PBMC	Clone	% of PBMC	Clone
CD2^+^	87.1 ± 3.3	No data	*Na*	*Na*
CD3^+^	90.9 ± 2.6	No data	*Na*	*Na*
CD4^+^	64.8 ± 5.9	*HB61A*	66.2 ± 12.3	*HB61A*
CD5^+^	92.0 ± 3.4	*HT23A*	91.8 ± 2.5	*HB19A*
CD8^+^	18.2 ± 3.3	*HT14A*	16.6 ± 2.8	*HT14A*
B lymphocytes				
Marker	% of PBMC	Clone	% of PBMC	Clone
CD19-like^+^	17.3 ± 2.5	*WS37*	*Na*	*Na*
CD21^+^	10.7 ± 5.2	No data	*Na*	*Na*
IgM^+^	17.0 ± 3.1	No data	11.3 ± 2.5	*1.9/3.2*

*N*, number of horses; *Na*, not applicable; PBMC; peripheral blood mononuclear cells.

**Figure 1:**
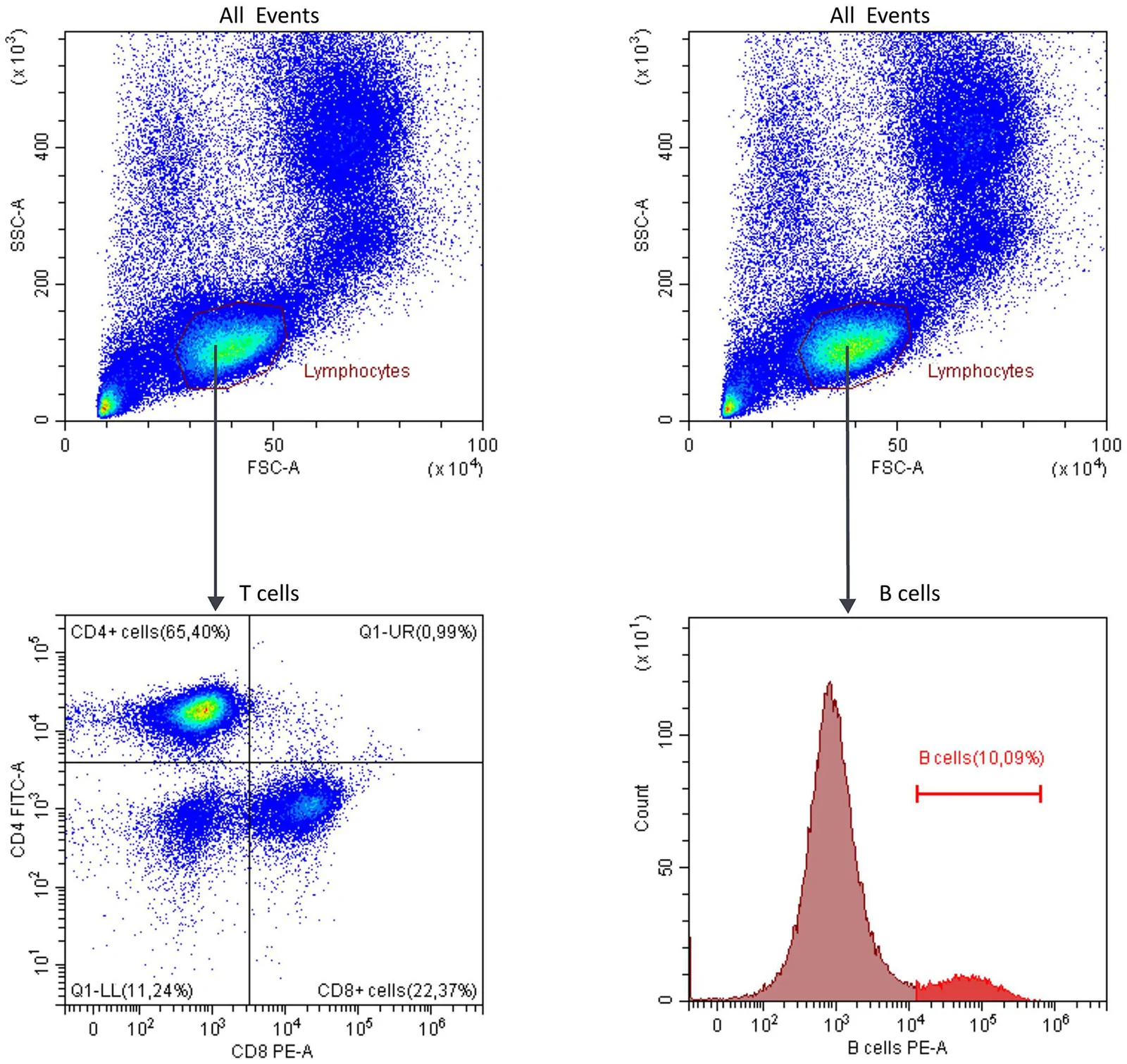
Peripheral blood mononuclear cells (PBMCs) were isolated from the blood of a healthy horse using Histopaque-1077 density gradient centrifugation. Lymphocytes were identified and gated based on their characteristic forward scatter (FSC) and side scatter (SSC) properties. The gated lymphocyte population was subsequently analyzed for the expression of CD4 and CD8 surface markers to distinguish helper (65.40%) and cytotoxic T-cell (22.37%) subsets. In parallel, B lymphocytes (10.09%) were identified using a pan-B cell antibody and visualized on a histogram. The following monoclonal antibodies were used for immunophenotyping: anti-equine CD4 (clone CVS4, FITC-conjugated), anti-equine CD8 (clone CVS8, PE-conjugated), and anti-equine pan-B (clone CVS36, PE-conjugated). The screening was performed using negative and isotype controls.

## 4 Clinical application of cells immunophenotyping in horses

### 4.1 Diagnosis of immunodeficiencies

Flow cytometry represents a valuable diagnostic modality for disorders in which the underlying pathology involves specific lymphocyte subpopulations and requires their quantitative assessment in peripheral blood. Immunophenotyping of peripheral blood lymphocytes (PBL) is a useful adjunctive technique in the diagnosis of immunodeficiency, particularly conditions associated with B cells, such as Common Variable Immunodeficiency (CVID) and Foal Immunodeficiency Syndrome, as well as transient CD4^+^ lymphopenia in young horses (26). CVID in horses is a rare, late-onset, non-familial immunological disorder characterized by depletion and/or functional impairment of B cells, leading to insufficient antibody production (27). Histopathological and immunohistochemical examinations typically demonstrate an absence of lymphoid follicles and B cells in primary and secondary lymphoid organs (28). Clinically, the disease is manifested by recurrent fever and infectious complications, most frequently involving the upper and/or lower respiratory tract, meningitis, cholangiohepatitis, infectious colitis, infectious dermatitis, and severe gastrointestinal parasitism (26, 27). In laboratory diagnostics, key abnormalities include a reduction in serum IgG concentration to < 10 g/L in two consecutive measurements and, in contrast to the situation in humans, the presence of B-cell lymphopenia in the majority of affected horses. In the case series reported by Felippe (27), encompassing 100 clinical cases, 63% of horses demonstrated < 6% B cells within the PBMC fraction, whereas the remaining 37% comprised animals with either normal or, paradoxically, increased B-cell percentages. Notably, in almost all affected horses, CD21^+^ cells exhibited low fluorescence intensity (CD21^low^), a feature likewise observed in human patients with this immunodeficiency (27). Some horses with CVID may also present with concurrent CD4^+^ T-cell lymphopenia, leading to a decreased CD4:CD8 ratio (< 2.0), which in some instances appears to be associated with inadvertent glucocorticoid administration (26). Prior to the 2024 summary by Felippe, flow-cytometric diagnostic findings had already been reported in several individual equine CVID cases, including animals with concomitant infections such as bacterial meningitis, chronic peritonitis, or neuroborreliosis (23, 29–31). All described patients exhibited a marked reduction in the proportion of B lymphocytes, which permitted an unequivocal diagnosis. In many cases, additional functional assays (e.g., proliferation assays) demonstrated decreased B-cell responsiveness to Pokewood Mitogen (PWM) and bacterial lipopolysaccharide (LPS) (29, 31). Owing to the current lack of knowledge regarding the mode of inheritance of this defect, immunological panel analysis (commercially available and including PBL immunophenotyping and quantitative determination of serum immunoglobulins) constitutes the cornerstone of diagnostic workup when CVID is suspected in horses. Another entity within the group of primary immunodeficiencies in horses characterized by B-cell lymphopenia is Foal Immunodeficiency Syndrome (FIS), also termed Fell Pony Foal Syndrome (FPS) (26). This disorder is a lethal hereditary condition affecting Fell Pony and Dales Pony horses (26). Clinically, affected foals are typically born without overt abnormalities and, within a few weeks, develop severe manifestations of non-regenerative anemia. Immediately postpartum, the proportion of B lymphocytes does not differ from reference values for healthy foals; however, within several weeks, B lymphocytes in affected foals progressively disappear, coinciding with the development of disseminated (multi-organ) infections (32, 33). The presence of B-cell lymphopenia represents a defining characteristic of the FIS phenotype (33). Additionally, the expression of MHC class II molecules on PBL may be reduced and/or may fail to exhibit the normal age-related increase observed in healthy foals, further suggesting aberrant lymphocyte development (33). However, Thomas et al. (34) reported no significant differences in MHC class II expression on the surface of B lymphocytes in affected foals. Flow cytometric analyses have not demonstrated T-cell deficiencies, showing normal distributions of CD4^+^ and CD8^+^ lymphocyte subsets, as well as normal lymphocyte proliferative responses to mitogenic stimulation in affected foals (35). The studies conducted by Thomas et al. (32, 34) confirmed the diagnostic utility of PBL immunophenotyping for FIS, but indicated that a definitive diagnosis should be established on the basis of postmortem examination or through genetic testing specific to Fell Pony and Dales Pony breeds. Another lymphocyte-related disorder described in horses is transient CD4^+^ lymphopenia. In some cases, foals show a delayed increase in lymphocyte counts, without documented lymphocytosis, accompanied by a delayed attainment of the expected proportion of CD4^+^ lymphocytes (< 50%) in peripheral blood, resulting in reduced CD4:CD8 ratios (< 2.0). Furthermore, this condition is frequently associated with a failure to up regulate MHC class II expression on lymphocytes, while MHC class II expression on monocytes remains unaffected (26).

### 4.2 Diagnosis of hematopoietic neoplasia

During diagnostic workup for suspected hematologic neoplasia in animals, flow cytometric immunophenotyping is routinely employed to address two principal questions: (1) whether the patient is affected by lymphoma and leukemia and, if so, (2) which specific lymphoma or leukemia subtype is present (36). Flow cytometry is particularly useful in the assessment of peripheral blood lymphocytosis or lymphadenopathy when cytologic evaluation alone is insufficient to reliably differentiate neoplastic from reactive lymphoid proliferations. Neoplasms of this type are relatively uncommon in horses and are generally associated with a poor prognosis. Analogous to human medicine, equine leukemias are classified as acute (acute lymphoid or myeloid leukemia–ALL/AML) and chronic (chronic lymphocytic leukemia – CLL). This distinction is based not on disease duration but on the maturation stage of the predominant neoplastic cell population: chronic forms are dominated by morphologically mature cells, whereas acute forms are characterized by immature blasts. In all neoplasm types, including lymphomas, the neoplastic process may arise from different hematopoietic lineages (37). CLL in horses typically occurs in older animals and is manifested by lymphocytosis, often accompanied by alterations in lymphocyte size (small to medium-sized cells) and concurrent cytopenias such as non-regenerative anemia and thrombocytopenia (26). Since the 1990s, several equine CLL cases have been reported, with 8 of these characterized using flow cytometric immunophenotyping. These studies indicate that equine CLL most frequently involves T helper cells (CD4^+^ cells) (38–41). A single case of B-cell CLL with concomitant hyperparathyroidism was diagnosed by immunophenotyping and described in a mule (42). Acute leukemias in horses most commonly comprise ALL and AML and may originate from various hematopoietic lineages. They are typically characterized by the presence of immature cells, non-regenerative pancytopenia, and frequent detection of blasts in peripheral blood. The clinical course of ALL in horses can resemble that of lymphoma, and the two entities may be difficult to distinguish, as both can infiltrate the bone marrow (although marrow infiltration is usually less pronounced in lymphoma). The diagnostic definition of ALL includes bi- or pancytopenia and the presence of >25% blasts in the bone marrow (36). In small animals (dogs and cats), a characteristic immunophenotypic hallmark of acute leukemia is the detection of CD34^+^ cells (a stem/progenitor cell marker) in peripheral blood (43). Expression of this marker on equine mesenchymal stem cells (MSCs) has also been documented and has been utilized in the diagnostic procedure of single equine case of ALL (44, 45). However, other authors indicate that equine MSCs derived from bone marrow are negative for this marker. Among equine ALL cases characterized by flow cytometry, B-cell ALL (B-ALL) has predominated; however, a single case originating from CD4^+^ T cell (T-ALL) has also been reported (40, 46, 47). Notably, in this T-ALL case, as many as 20% of neoplastic cells exhibited a CD3^−^CD4^+^ immunophenotype, negatively for myeloid cell marker (CD13), which is considered aberrant (40). Among AML in horses, the most frequently described subtypes are AML-M4 (myelomonocytic) and AML-M5 (monocytic), according to the classification of the Animal Leukemia Study Group (ALSG) (46, 48). By accepted criteria, AML-M4 is diagnosed when >20% of tumor cells are neutrophils or monocytes, with >20% blasts in blood and bone marrow, and >20% of bone marrow cells belonging to the monocytic lineage (48). AML-M5 is defined by the presence of >80% monocytic lineage cells (monoblasts and/or promonocytes) among non-erythroid cells in the bone marrow (48). To date, only a few equine AML-M4 and AML-M5 cases have been diagnosed using flow cytometry. In this case, evaluation included plotting of FSC and SSC, as well as immunophenotyping with markers such as CD172a (myeloid cells–clone DH59B), CD14 (monocytes–clone 105), CD34 (immature progenitor cells–clone 1H6), and CD163 (clone LND68A) and Iba-1/AIF-1 (macrophages) (45). Lymphomas represent the most prevalent hematopoietic neoplasms in horses, accounting for up to 3% of all neoplastic lesions. They are typically classified into multicentric, alimentary, mediastinal, and cutaneous forms. Leukemia associated with lymphoma has been reported only sporadically (49). Owing to the characteristic morphology of these lesions (frequently nodular and involving lymph nodes), immunophenotyping in the diagnostic workup of equine lymphomas is most commonly performed on biopsy specimens using immunohistochemistry (50). In the study by Meyer et al. (49), flow cytometric analysis was applied to fresh neoplastic effusion samples, enabling characterization of the predominant lymphocyte subpopulations within the material, which included five T-cell lymphomas and one B-cell lymphoma. The study by de Bruijn et al. (51) further demonstrated the utility of flow cytometry in the diagnosis of cutaneous malignant lymphoma. Cytometric evaluation of tumor cells obtained via fine-needle aspiration (FNA) indicated that cutaneous malignant lymphomas were composed predominantly of CD4^+^ and CD8^+^ T-lymphocytes (51). Concurrently, immunophenotyping of peripheral blood leukocytes revealed only minor deviations from reference values. Relative to clinically healthy horses, there was a reduction in the overall proportion of cells within the lymphocyte gate, and in three horses with the cutaneous form of malignant lymphoma an increased number of CD4^+^ cells was detected within the monocyte gate (51). Although immunohistochemistry remains more widely available and cost-effective, adjunctive flow cytometric analysis, particularly when combined with FNA sampling, can yield substantial additional information regarding the biological behavior and immunophenotype of the neoplasm, a diagnostic approach already routinely implemented in canine and feline oncology (43). From a flow cytometric standpoint, the immunophenotypic characterization of neoplastic B cells is particularly noteworthy, as accumulating evidence indicates that these cells frequently fail to express the full repertoire of canonical surface markers, a phenomenon that is especially pronounced upon differentiation into plasma cells. In the five analyzed cases of B-cell lymphoma/leukemia, the neoplastic populations were predominantly positive for IgG1 or IgG4/7, while exhibiting low-level expression of CD19-like, CD21, or IgM (markers considered characteristic of the B-cell lineage in horses), in association with T-cell lymphopenia (52). All examined horses displayed monoclonal gammopathy. The lack of CD19 expression in equine cases of monoclonal gammopathy has already been documented in previous reports (52, 53).

## 5 Clinical application of BALF immunophenotyping in horses

Bronchoalveolar lavage fluid (BALF) cytology is regarded as the diagnostic gold standard for equine asthma (EA), a disease previously termed chronic obstructive pulmonary disease (COPD) or recurrent airway obstruction (RAO) (54). This disorder is characterized by non-infectious pulmonary inflammation, typically dominated by neutrophilic infiltration, accompanied by increased mucus production and bronchoconstriction (54). The pathogenesis of EA has not yet been conclusively elucidated, but it involves immune mechanisms that lead to neutrophil infiltration and pro-inflammatory effects (54). The reference method for establishing the diagnosis is cytological evaluation of BALF (55). For each specimen, a minimum of 500 cells should be differentially counted, and the relative proportions of lymphocytes, macrophages, neutrophilic granulocytes (polymorphonuclear cells, PMN), eosinophilic granulocytes and metachromatic mast cells (using Diff-Quick-stained slides or toluidine blue-stained slides) should be recorded. In healthy horses, BALF cytology is expected to be dominated by macrophages (50%−70%) and lymphocytes (30%−50%), with only low percentages of neutrophils, mast cells, and eosinophils (56). On the basis of several studies employing a 250-mL lavage volume, proposed reference values for BALF cytology in clinically healthy controls include a total nucleated cell count ≤ 530 cells/μL, neutrophils ≤ 5%, eosinophils ≤ 1%, and metachromatic mast cells ≤ 2% (54). Other published data suggest slightly different normal limits, namely: ≤ 8% neutrophils, ≤ 0.5% eosinophils, and ≤ 5% mast cells (57). To date, flow cytometry has not been routinely applied to equine BALF analysis because of its relatively high cost and restricted accessibility. Nonetheless, substantial progress has been made in recent years, allowing advanced immunophenotyping of lymphocyte subpopulations and performance of functional assays (including the assessment of apoptosis, oxidative burst activity, and phagocytic capacity) in comparative studies of control horses and animals affected by different clinical phenotypes of EA (58–60). The conventional SSC vs. FSC (Area) plot allows for presumptive gating of eosinophils, alveolar macrophages, neutrophils, lymphocytes, and monocytes; an example is reported in Supplementary Figure S1 in Supplementary File 1. However, their full identification is possible only with the use of appropriate markers.

In human medicine, the monocyte population is commonly described as pulmonary monocyte-like cells (PMLCs) (61). Phenotyping of lymphocytes in equine BALF showed the predominance of CD8^+^ cells within the lymphocyte population and enabled the proposal of reference ranges for healthy horses: B cells (surface IgG^+^−5.6%, SD = 1.34), CD3^+^ (clone F6G-3−88.3%, SD = 2.81), CD4^+^ (clone CVS4−31.6%, SD = 6.8), CD8^+^ (46.0%, SD = 5.8), and a CD4:CD8 ratio of 0.72 (SD = 0.23) (62). A detailed cytometric characterization of BALF has been provided by Gressler et al. (57). Immunophenotyping was conducted using the following antibodies: CD8 (clone CVS8), CD4 (clone HB61A), CD14 (clone 105), CD16 (clone 9G5), CD79a (clone MH47), CD154 (clone 5C8), neutrophils (clone DH24A), MHCII (clone cz11), as well as antibodies directed against IgG, IgM, IgE, and selected pro-inflammatory cytokines (57). These analyses demonstrated marked immunophenotypic differences between BALF and PBMC samples, including: (a) a predominance of CD8^+^ T lymphocytes in BALF, in contrast to the predominance of CD4^+^ T lymphocytes in PBMCs in lymphocytes population; and (b) co-expression of CD14 and CD16 on alveolar macrophages, whereas CD14 was expressed in the absence of CD16 on circulating monocytes (57). The alveolar macrophage population was additionally characterized using CD163 (clone Ber-Mac3) and CD206 (clone 3.29B1), consistent with an anti-inflammatory or alternatively activated phenotype and clearly distinguishing these cells from blood monocytes (63, 64). In the study by Gressler et al. (57), a subpopulation of CD16^−^IgE^+^ cells was identified for the first time as mast cells. Within the lymphocyte compartment, all Ig^+^CD4^−^CD8^−^ cells in PBMCs were CD79a-positive, whereas their counterparts in BALF lacked CD79a expression. This finding suggests that these BALF cells may represent unconventional T lymphocytes, natural killer (NK) cells, or other innate lymphoid cell subsets that could not be further delineated with the marker panel employed (57). Furthermore, assessment of the T helper cell activation marker CD154 (CD40L) and intracellular cytokine production revealed a comparable overall degree of T cell activation between groups, but with an overrepresentation of IL-17A^+^ cells in horses diagnosed with EA (57). In the context of investigations into the pathogenesis of EA, particular attention was directed to the presence of regulatory T cells in BALF and peripheral blood. The analysis employed an extracellular CD4 marker (clone CVS4 or HB61A) in combination with intracellular staining using an anti-FoxP3 antibody (clone FJK-16s) (65–67). Henriquez et al. (65) broadened this immunophenotypic profile to include CD25 staining, and, together with the findings of Hamza et al. (68), demonstrated that the regulatory T-cell phenotype in horses is CD4^+^CD25^+^FoxP3^+^ or CD4^+^CD25^high^. This immunophenotypic pattern was observed in PBL and BALF cells from both non-exposed horses and horses experimentally or naturally exposed to antigen (68). Consistent with the previously described analyses of PBL, the BALF analyses also demonstrated age-dependent maturation of peripheral lymphocyte subsets (16, 69). Both studies characterized the cellular composition of BALF in foals from birth up to 10 or 16 weeks of age, with a primary focus on the following lymphocyte populations: CD5 (clones CVS5 or HT23A), CD4 (clones CVS4 or HB61A), CD8 (clones CVS8 or HT14A), B cells (anti-equine IgG or clone B29A), CD44 (clone CVS18), MHC class I (clones CZ3 or H42A), MHC class II (clone TH14B), and granulocyte–monocyte EqGM1 (clone DH59B) (16, 69). The findings revealed a lower proportion of CD5^+^ cells in foals compared with their dams, as well as an age-related shift in the CD4:CD8 ratio from 1.26 to 0.78 (with a ratio of 0.66 in adults) (69). Similar to the situation in adults, macrophages were the predominant cell population in BALF from birth in foals, whereas the relative frequencies of lymphocyte subpopulations increased gradually. This was characterized by a rise in CD5^+^ cells (including CD4^+^, CD8^+^, and B cells) at approximately 2 months of age, followed by a gradual decline thereafter (16). Although the development of the respiratory immune system parallels changes observed in peripheral blood, processes such as cellular proliferation, activation, and migration may proceed at a slower pace in the respiratory tract. Consequently, the pulmonary microenvironment may remain particularly vulnerable to pathogenic challenge before the immune system has fully matured (16). The integration of imaging with advanced flow cytometry also permits the quantitative evaluation of airway epithelial cells in BALF samples (Supplementary Figure S2 in Supplementary File 2).

## 6 Clinical application of body cavity fluids immunophenotyping in horses

Flow cytometric evaluation of peritoneal and pleural effusions is well established in human medicine. In equine patients, however, only a single study has been published to date that characterizes the cellular composition of exudates and transudates from the peritoneal and pleural cavities. In that investigation, 12 transudates (defined by a total nucleated cell count < 5 × 10^9^/L), predominantly associated with colic, fever of unknown origin, or lymphoma, and 6 exudates secondary to colic or aseptic/septic peritonitis were analyzed (70). Immunophenotyping was performed using the following markers: CD3 (clone CD3-12, an intracellular T-cell marker), CD4 (clone CVS4), CD8 (clone CVS8), and CD21 (clone CA2.D6) (70). The results demonstrated a predominance of T lymphocytes in both transudative and exudative effusions, with a high proportion of CD4^+^ cells and a relatively low number of B lymphocytes (70). Nevertheless, the absence of a healthy control group limits the interpretability of these findings and constrains their direct application to clinical diagnostic decision-making.

## 7 The use of flow cytometry in the examination of semen

The assessment of semen quality is essential for evaluating stallion fertility and breeding potential, providing valuable information for predicting fertilization success under both natural mating conditions and assisted reproductive technologies. In this context, flow cytometry is a method that enables rapid, multiparametric, and objective evaluation of sperm quality, complementing conventional semen assessment methods such as concentration measurement, Computer-Assisted Sperm Analysis (CASA), and microscopic evaluation of sperm morphology. Its main advantage lies in the ability to analyze functional sperm parameters that are not detectable in routine semen diagnostics, such as plasma membrane integrity, mitochondrial membrane potential, acrosome status, ROS production, and DNA integrity, thereby allowing a more precise assessment of sperm fertilizing potential. Compared with many clinical applications of flow cytometry in equine immunology, oncology, and internal medicine, the literature on stallion semen is relatively extensive, as flow cytometric assessment of stallion spermatozoa has been investigated for several decades. This may partly reflect the use of broadly applicable fluorescent dyes and probes, whereas immunophenotyping depends on the availability and validation of equine-specific antibodies. Nevertheless, many studies using flow cytometry in stallion semen evaluation remain methodological in nature; however, in selected research and clinical centers, flow cytometry-based methods, alongside standard semen analysis, also form part of extended diagnostic protocols. Examples of flow cytometric assays used to assess sperm functional parameters are summarized in Table 3.

**Table 3:** Examples of the most commonly used flow cytometric assays for the assessment of stallion sperm functional parameters.

Parameter assessed	Method	Example references
Cell viability/membrane integrity	Propidium Iodide (PI) staining	(101, 102)
	Ehidium Homodimer-1 (EtHD) staining	(79)
	SYTOX staining	(80, 97, 116, 119)
	SYBR-14 and PI co-staining	(71, 73, 75, 81–83, 98, 103–105, 173)
	Fixable amine dyes staining	(73, 93, 97, 110, 119)
Apoptosis	Annexin V and PI co-staining	(81, 84)
	Yo-Pro-1 and EtHD/PI co-staining	(71, 77–87, 89, 90)
	Caspase-3/7 activation: -FITC-DEVD-FMK -CellEvent™ Caspase-3/7 Green Detection Reagent	(80, 81, 87, 93)
	Pan-caspase activation: -FITC-VAD-FMK	(79, 85, 86, 90)
Mitochondrial membrane potential	JC-1 staining	(71, 73, 77, 79–81, 83, 85, 86, 88–90, 93, 94, 97, 103–107)
	Rhodamine 123 staining	(101, 102)
	Tetramethylrhodamine methyl ester (TMRM) staining	(94)
Reactive oxygen species (ROS) and oxidative stress	CellROX staining	(77, 105, 106, 118)
	MitoSOX staining	(95, 97, 117–119)
	Dihydroethidium (DHE) staining	(71, 87, 97, 98, 104, 116, 117)
	OxyDNA assay	(80, 97, 106)
Lipid peroxidation	BODIPY 581/591 C11	(81, 82, 84, 86, 89, 90, 117)
	Anti-4-hydroxynonenal (4-HNE) antibody	(93, 97, 110, 119, 122, 123)
Chromatin integrity/DNA damage	Sperm chromatin structure assay (SCSA; acridine orange staining)	(71, 97, 98, 104, 106, 134, 135, 174–176)
Acrosome status/acrosome integrity	Peanut agglutinin (PNA) conjugated with a fluorophore staining	(71, 82, 83, 97, 102, 104, 105, 118)
Morphological and multiparametric cellular analysis	Imaging flow cytometry	(94, 116, 142)

The listed assays are the most frequently used in both published studies and commercial laboratories. References are provided as representative examples from the literature.

The increasing application of flow cytometry is driven by the need for more precise sperm evaluation for assisted reproductive technologies (ARTs), particularly artificial insemination (AI) and intracytoplasmic sperm injection (ICSI), which are increasingly used in horse breeding. In this regard, flow cytometry enables objective assessment of functional changes in spermatozoa induced by cooling, cryopreservation, and semen transport, allowing identification of stallions and ejaculates with varying resistance to these preservation procedures. This is particularly important in horses, as stallion spermatozoa generally exhibit reduced post-thaw viability and functional competence compared with those of other species, as well as considerable inter-stallion variability in cryotolerance, commonly classified as “good” and “poor” freezers (71, 72).

### 7.1 Evaluation of sperm viability and apoptosis

One of the primary functional parameters assessed by flow cytometry is sperm viability. This method uses membrane-impermeable fluorescent dyes that are weakly fluorescent until they bind to DNA or RNA. In live cells, intact membranes prevent dye entry, whereas in dead cells the dyes penetrate, bind to nucleic acids, and fluoresce upon laser excitation. Propidium iodide (PI) is most commonly used, but alternatives include 7-aminoactinomycin D (7-AAD), Ethidium Homodimer-1 (EtHD), DRAQ7^TM^ and SYTOX^TM^ dyes. However, these dyes are not suitable for fixation protocols, therefore fixable viability dyes available in multiple fluorescence channels have been developed (e.g., Zombie Fixable Viability^TM^ Kit, LIVE/DEAD^TM^ Fixable Dead Cell Stain Kit). These dyes bind to cellular amines before fixation, enabling reliable post-fixation analysis. Their performance has been shown to be comparable to that of the conventional PI/SYBR-14 assay, including in stallion sperm (73). This approach enables transport and delayed analysis of fixed samples, which may be particularly important in multicenter studies or when immediate cytometric analysis is unavailable. Currently, stallion sperm viability is most commonly assessed in flow cytometry using combinations of membrane-permeable and membrane-impermeable DNA dyes, which also allow discrimination of unstained debris. A widely used combination is SYBR-14, which stains live cells green, and PI, which penetrates dead cells and stains them red (74, 75). An important advantage of this co-staining is that both dyes are excited by a 488 nm blue laser, available in most flow cytometers. Alternatively, Hoechst 33342 combined with PI can also be used for the same purpose (76, 77) but requires a violet or ultraviolet laser, which may not be available in all instruments. In SYBR-14/PI staining, three sperm populations can be identified: viable (green fluorescence), dead (red fluorescence), and moribund (showing both green and red fluorescence). Ready-to-use commercial sperm viability kits containing SYBR-14 and PI are available together with detailed staining instructions (e.g., the LIVE/DEAD^TM^ Sperm Viability Kit; Thermo Fisher Scientific).

SYBR-14/PI staining, however, does not detect early apoptotic changes, as membrane integrity is preserved during the initial stages of apoptosis. Therefore, this process is commonly assessed using Annexin V conjugated to a fluorophore, usually Alexa Fluor 488 or fluorescein isothiocyanate (FITC), combined with PI. During early apoptosis, phosphatidylserine translocates to the outer leaflet of the plasma membrane and can therefore be bound by Annexin V. In this staining, four sperm populations can be distinguished: viable (Annexin V^−^/PI^−^), early apoptotic (Annexin V^+^/PI^−^), late apoptotic or secondary necrotic (Annexin V^+^/PI^+^), and dead/necrotic (Annexin V^−^/PI^+^) (78). Early membrane alterations in stallion spermatozoa can also be evaluated using Yo-Pro-1 with EtHD or PI (71, 77, 79–90). Yo-Pro-1 is a membrane-impermeant dye; however, during early apoptosis, changes in membrane permeability allow its selective uptake (91). Studies have shown that the Yo-Pro-1 assay detects stallion sperm quality differences not identified by standard SYBR-14/PI staining and correlates more strongly with sperm kinematics (81). Using this staining, spermatozoa can be classified into four subpopulations: viable (unstained) cells, early apoptotic (Yo-Pro-1 positive; green fluorescence), dead (EtHD or PI positive; red fluorescence) and late apoptotic (double-stained; green and red fluorescence).

Another approach for detecting apoptosis is the assessment of caspase activity. Caspases are key mediators of programmed cell death, initiating and executing the proteolytic cascade that leads to controlled cellular disassembly (92). In studies on stallion spermatozoa, FITC-VAD-FMK has been used as a fluorescent probe to detect caspase activity. The VAD sequence (Val-Ala-Asp) enables irreversible binding of the probe to active caspases by mimicking their natural substrates, whereas the fluoromethyl ketone (FMK) group covalently blocks the catalytic site of the enzyme. The FITC fluorochrome provides the fluorescent signal, allowing apoptotic spermatozoa with active caspases to be identified by flow cytometry. When combined with EtHD or PI staining, this method enables the identification of four sperm subpopulations similarly to Annexin V/PI staining (79, 85, 86, 90). A similar reagent, FITC-DEVD-FMK, contains the amino acid sequence DEVD (Asp-Glu-Val-Asp), which is specifically recognized by caspases 3 and 7. These caspases are key executioner enzymes and are widely regarded as principal markers of apoptosis, as their activation indicates an irreversible stage of cell death. FITC-DEVD-FMK has also been successfully applied in studies evaluating apoptosis in equine spermatozoa (81, 93). Another assay used to detect caspase-3/7 activity in stallion spermatozoa is the CellEvent^TM^ Caspase-3/7 Green Detection Reagent (Invitrogen). Like FITC-DEVD-FMK, it contains the DEVD peptide sequence recognized by active caspases 3 and 7. Cleavage of this substrate releases a DNA-binding dye that produces bright green fluorescence in apoptotic cells (80, 87).

### 7.2 Assessment of sperm mitochondrial function

In mammalian spermatozoa, mitochondria are localized in the midpiece (94) and generate adenosine triphosphate (ATP) required for sperm motility and contribute to the regulation of reactive oxygen species (ROS) production (95), apoptosis (79, 90), and calcium homeostasis (95). In stallion spermatozoa, mitochondrial oxidative phosphorylation (OXPHOS) contributes to ATP production required for the maintenance of membrane integrity, whereas sperm motility is supported by both OXPHOS and glycolysis (77, 90, 96–98). Accordingly, mitochondrial function has been associated with sperm motility parameters (96, 97).

ATP synthesis via oxidative phosphorylation is driven by the mitochondrial membrane potential (ΔΨ*m*), an electrochemical proton gradient generated by the respiratory chain across the inner mitochondrial membrane (99). Studies in stallions have shown that cooling and cryopreservation reduce ΔΨ*m* and impair oxidative phosphorylation, resulting in increased ROS production and reduced ATP synthesis (71, 79, 100). Therefore, the assessment of mitochondrial function is considered a key element in evaluating sperm quality and fertilizing potential.

In stallion sperm analysis, MitoTracker^TM^ dyes are primarily used for mitochondrial labeling and localization, whereas ΔΨ*m* is typically assessed using cationic dyes like Rhodamine 123 (101, 102), tetramethylrhodamine methyl ester (TMRM) (94), or JC-1 (71, 73, 77, 79–81, 83, 85, 86, 88–90, 93, 94, 97, 103–107). Rhodamine 123 and TMRM accumulate in mitochondria in a ΔΨ*m*-dependent manner, resulting in increased fluorescence intensity (green and orange-red, respectively) with increasing membrane potential. In contrast, JC-1 is a ratiometric dye that forms orange-red fluorescent aggregates at high ΔΨ*m* and green fluorescent monomers at low ΔΨ*m*, with the red-to-green fluorescence ratio reflecting mitochondrial membrane potential.

### 7.3 Assessment of reactive oxygen species (ROS) in spermatozoa

ROS comprise both oxygen-derived free radicals, such as superoxide (${\text{O}}_{2}^{-}•$), the hydroxyl radical (•OH) and lipid peroxyl radicals (LOO•), as well as non-radical oxidants such as hydrogen peroxide (H_2_O_2_). At physiological concentrations, ROS contribute to several key events of fertilization, including sperm capacitation and the acrosome reaction (108), while excessive production of ROS leads to oxidative stress and damage to, among others, sperm plasma membranes, mitochondria and DNA (109). Generation of ROS in stallion semen may be due to the activity of L-amino acid oxidase (LAAO), which catalyzes the production of H_2_O_2_ from aromatic amino acids. Its activity is especially enhanced in the presence of dead sperm (110). However, electron leakage from the mitochondrial electron transport chain is considered the main intracellular source of ROS in spermatozoa (111). Cryopreservation has been shown to increase ROS production (71, 112) through several mechanisms, including the metabolism of aromatic amino acids present in egg yolk-based extenders by LAAO (110), the loss of antioxidant protection provided by seminal plasma removed during semen processing (113), and mitochondrial damage induced by osmotic stress (114). ROS levels in stallion sperm should be interpreted with caution because their high mitochondrial activity may contribute to physiological ROS generation (98). Moreover, some studies have even reported a positive association between ROS levels and sperm motility (97). The unique aspects of redox regulation and oxidative stress in stallion spermatozoa have been reviewed in detail by Peña et al. (115). In stallion sperm research, dihydroethidium (DHE) is commonly used to assess intracellular ROS production, particularly superoxide anion generation (71, 87, 97, 98, 104, 116, 117). In the presence of ${\text{O}}_{2}^{-}•$, DHE is oxidized to 2-hydroxyethidium, which binds to DNA and emits red fluorescence upon excitation with a 488 nm laser. For the detection of intracellular ROS in stallion spermatozoa, commercially available fluorescent probes, such as the frequently used CellROX^TM^ reagent (Invitrogen), are also applied (77, 105, 106, 118). These probes operate on a similar principle, although the excitation and emission wavelengths depend on the specific probe used. Depending on the probe, fluorescence may occur either directly upon oxidation or increase upon binding to nucleic acids, as does DHE. To specifically evaluate mitochondrial superoxide production in stallion spermatozoa, MitoSOX^TM^ indicators (Invitrogen) can be used (95, 97, 117–119). MitoSOX^TM^ is a mitochondria-targeted hydroethidine derivative that selectively accumulates within mitochondria due to the presence of a triphenylphosphonium moiety. Upon oxidation by mitochondrial ${\text{O}}_{2}^{-}•$, it is converted into fluorescent oxidation products which bind to nucleic acids. The resulting fluorescence is detected as either green or red fluorescence, depending on the specific MitoSOX^TM^ variant used. In flow cytometry, ROS probes are often combined with viability dyes to exclude dead spermatozoa from analysis.

Some studies have also investigated the effects of ROS on specific cellular structures in spermatozoa. Oxidative DNA damage was detected using the OxyDNA assay kit, which utilizes a fluorescence-conjugated antibody recognizing 8-oxoguanine (80, 97, 106). The sperm plasma membrane is also particularly susceptible to oxidative damage due to its high content of polyunsaturated fatty acids (120, 121). Lipid peroxidation can be detected using C_11_BODIPY^TM^ 581/591, a fatty acid analog which incorporates into the plasma membrane (81, 82, 84, 86, 89, 90, 117). This fluorescent probe, upon oxidation by ROS, exhibits a change in fluorescence emission from red to green. Another important indicator of oxidative stress in stallion spermatozoa is 4-hydroxynonenal (4-HNE), a by-product of lipid membrane peroxidation (93, 97, 110, 117, 119, 122, 123). As a highly reactive aldehyde, 4-HNE induces DNA damage, forms adducts with proteins, and triggers a significant increase in mitochondrial and cellular ROS production, ultimately resulting in the perturbation of cell integrity and function (117, 122). Detection of 4-HNE is based on the use of specific primary antibodies against 4-HNE, followed by fluorochrome-conjugated secondary antibodies and fluorescence analysis.

### 7.4 Assessment of acrosome integrity

The acrosome is a cap-like organelle located over the anterior portion of the sperm head that contains enzymes required for penetration of the oocyte's zona pellucida, thereby enabling fertilization. Therefore, both damaged acrosomes and premature acrosome reactions are associated with reduced fertilizing capacity of spermatozoa by causing the untimely release or loss of these enzymes. The structure, function and clinical relevance of the stallion sperm acrosome are reviewed in detail by Hernández-Avilés et al. (124). In fluorescence-based methods, including flow cytometry, acrosome integrity is assessed using fluorescently labeled lectins that bind acrosomal membrane components, such as *Pisum sativum* agglutinin (PSA) (125) and *Arachis hypogaea* agglutinin (PNA) (126). The most commonly used is PNA which specifically binds to β-galactose residues on the outer acrosomal membrane. It should be noted that the interpretation of fluorescence signal is closely dependent on the sperm preparation protocol used. When analyzing live sperm to which PNA is added without prior membrane permeabilization, the lectin cannot access the interior of the intact acrosome. As a result, sperm with an intact acrosome remain PNA-negative, while sperm with a damaged acrosome or those that have undergone an acrosome reaction become PNA-positive because PNA can penetrate the damaged membranes and bind to the exposed acrosomal structures (127). This method is a more commonly used in flow cytometry-based studies, including stallion semen (71, 82, 83, 97, 102, 104, 105, 118). A different staining pattern is observed when spermatozoa are fixed and permeabilized, allowing PNA to access intracellular acrosomal structures. Consequently, sperm with an intact acrosome are PNA-positive because the lectin can enter the cell and bind to the glycoconjugate-rich structures of the acrosome, whereas sperm with a damaged acrosome or those that have undergone an acrosome reaction exhibit a weaker fluorescence or are PNA-negative because they have lost some or all of their acrosomal contents (128). This approach is used predominantly in fluorescence microscopy studies, although it has also been described in some flow cytometric protocols (129). Acrosome evaluation is routinely combined with a viability dye (fixable or non-fixable, depending on the protocol used) to differentiate between true acrosome loss in viable spermatozoa and necrotic acrosome loss resulting from the loss of cell viability (127).

### 7.5 Sperm DNA fragmentation

Sperm DNA fragmentation (sDF), defined as single- or double-strand breaks in nuclear DNA, is an important marker of semen quality that may be independent of routinely assessed parameters such as sperm count, morphology, and motility (130). Although elevated sDF levels are associated with reduced embryo quality and lower pregnancy success rates, sDF assessment is not included in routine semen analysis and is mainly performed in selected clinical or research settings in both humans (131) and animals (132).

Sperm DNA integrity can be assessed using different methods, most of which are based on microscopic evaluation of sperm. Sperm Chromatin Structure Assay (SCSA), on the other hand, is based on flow cytometry and therefore allows for the rapid analysis of large numbers of cells (133). In SCSA, the semen sample, either fresh or frozen, is treated with a low-pH detergent solution that induces controlled DNA denaturation at sites susceptible to strand breaks and chromatin instability. Subsequently, the sample is neutralized and stained with acridine orange (AO). This metachromatic dye intercalates into DNA and, upon excitation with blue laser light (488 nm), emits green fluorescence when bound to double-stranded DNA and red fluorescence when bound to single-stranded DNA, reflecting DNA denaturation or strand breaks. Based on individual cellular fluorescence, a cell-level DNA Fragmentation Index (DFI) is calculated for each sperm as the ratio of red to total (red plus green) fluorescence. Consequently, the percentage of cells exceeding a specific threshold determined by a reference control sample is quantified, yielding the final %DFI value, which represents the proportion of abnormal sperm within the analyzed population (130).

Meta-analyses of several techniques for assessing sDF in animals have shown that SCSA has one of the strongest associations with fertility, indicating a statistically significant relationship between sDF and reproductive outcomes (132). In stallions, higher levels of sDF have been associated with reduced fertility, and SCSA has been shown to be a useful method for assessing sperm quality in both artificial insemination and natural mating (134). Additionally, studies have demonstrated that SCSA can be used to assess changes in sperm DNA integrity after exposure to different extenders and cooling conditions (135). For the assessment of DNA fragmentation to be more widely applied and useful in semen quality evaluation, it is clinically important to establish relevant threshold values. Given that several sDF evaluation methods exist, threshold values should be established and validated for the specific method and laboratory. In stallions, Evenson (133), based on SCSA findings reported by Kenney et al. (134), suggested a value of approximately 28% as a potential %DFI threshold for stallion fertility. However, this value should be considered context-dependent (i.e., dependent on the assay protocol, laboratory procedures, and study population) and should not be regarded as a universal cutoff value for stallions.

It should also be noted that AO staining in SCSA enables assessment of sperm chromatin maturity through the High DNA Stainability (HDS) index (formerly designated as high green fluorescence; HGRN). Spermatozoa with high HDS values exhibit abnormal nuclear protein composition and, consequently, incomplete chromatin condensation, resulting in increased accessibility of double-stranded DNA to the dye. Although DNA fragmentation is not present, insufficient chromatin packaging may compromise fertilizing potential (136). Information regarding HDS in the literature mainly concerns humans (137), whereas veterinary studies are less common and have primarily focused on bulls and boars (138). To date, information on HDS in horses remains very limited, therefore, further research is necessary to establish reliable threshold values and to verify the utility of this parameter for fertility assessment. This is particularly relevant given that, although in humans an HDS value below 15% is commonly referenced as normal in humans (139), some studies question its usefulness in semen quality evaluation (140, 141).

### 7.6 Imaging flow cytometry in evaluation of stallion spermatozoa

In recent years, imaging flow cytometry (IFC) has emerged as a valuable tool for sperm analysis, enabling both high-throughput quantitative assessment of large numbers of cells and visualization of their intracellular structures. While the examples presented below highlight its application in stallion sperm analysis, the technique can also be extended to a wide range of other sperm parameters, including those traditionally evaluated by flow cytometry or by bright-field and fluorescence microscopy. Its main advantage is the ability to combine large-scale quantitative analysis with simultaneous morphological imaging at the single-cell level.

In stallion semen, it has so far been applied to investigate the relationship between sperm morphological abnormalities and intracellular ROS production (116). In another study, the authors used imaging flow cytometry to demonstrate distinct subcellular localizations of the three lactate dehydrogenase (LDH) isoforms (A, B, and C) in stallion spermatozoa (142). This type of cytometry was also used after the TMRM assay to enable visualization and quantification of mitochondrial activity and to confirm that the fluorescence signal was localized to the mitochondrial midpiece in stallion spermatozoa (94). In addition, imaging flow cytometry allows identification of stallion spermatozoa exhibiting specific defects, including abnormal head, abnormal midpiece, abnormal tail, proximal cytoplasmic droplet, distal cytoplasmic droplet, and coiled tail. The authors further confirmed the reliability of imaging flow cytometry by showing that the percentage of spermatozoa classified as morphologically normal using this method closely matched the percentage determined by traditional light microscopy (116).

### 7.7 Flow cytometric sex sorting of stallion spermatozoa

Another quite different application of flow cytometry is spermatozoa sex sorting. Sex-sorted semen can be obtained using various biotechnological techniques that distinguish and separate sperm carrying X or Y chromosomes to produce offspring of the desired sex. However, flow cytometry-based sex sorting, which exploits the fact that X-bearing sperm contain approximately 2.8%−4% (~3.7% in stallions) (143) more DNA than Y-bearing sperm, currently provides the highest accuracy. In the method developed by Johnson et al. (144), sperm are stained with Hoechst 33342 and individually pass through a UV laser beam. Because X-bearing sperm contain more DNA, they emit stronger fluorescence than Y-bearing sperm, allowing their separation into distinct fractions.

Flow cytometric cell sorting, also known as fluorescence-activated cell sorting (FACS), is an extension of conventional flow cytometry that enables the physical separation of cells. Currently, flow cytometry systems equipped with cell-sorting capability typically use electrostatic droplet sorting to separate cells based on their fluorescence or light-scattering properties. The stream of cell suspension is broken into microdroplets containing single cells, which are then electrically charged and deflected into separate collection tubes (145). It is important to note that standard cell-sorting cytometers found in many research laboratories are usually not optimized for sperm sorting, as high-speed sorters with appropriate nozzle configurations, settings, and buffer systems are required, some of which are patented (143). Moreover, although flow cytometry can typically sort up to approximately 8,000 sperm per second (146), the relatively low number of straws produced per hour makes sperm sexing not only costly but also time-consuming.

Semen sexing is widely used, particularly in cattle reproduction. Flow cytometry remains the only reliable method for separating X- and Y-chromosome-bearing spermatozoa, achieving sorting purities and female calf birth rates of up to 95%, as demonstrated by commercial products such as SexedULTRA 4M™ (147). In horses, however, there is currently no widely available commercial supply of sexed stallion semen. This is mainly due to several characteristics of stallion sperm that reduce sorting efficiency. Sperm sorting primarily depends on the difference in DNA content between X- and Y-bearing sperm and on sperm head morphology. Larger, flatter, and more regular sperm heads facilitate hydrodynamic alignment in the flow stream, enabling more precise separation. Although the difference in DNA content between X- and Y-bearing sperm in stallions is similar to that observed in bulls, the smaller surface area of the stallion sperm head results in a significantly lower sorting index (head profile area, μm^2^ x X–Y DNA difference, %) than in cattle (59 vs. 131, respectively) (148).

During sex sorting, sperm cells are exposed to various stressors that reduce the fertilizing ability of sorted semen. Mechanical stress occurs during FACS, where sperm are forced under pressure through narrow channels (149). In addition, fluorescent dye staining, laser irradiation, and changes in the composition of the surrounding medium may induce further biochemical and chemical stress (150, 151). Stallion sperm appear to be more sensitive to these factors than, for example, bull sperm. Sex sorting has been shown to impair stallion sperm quality, reducing motility, velocity parameters, ATP content, mitochondrial membrane potential, and genomic integrity, while increasing the proportions of dead and apoptotic cells, ROS production, and DNA fragmentation. The observed DNA damage has been attributed largely to oxidative stress (84, 106). There are also additional challenges in equine semen sorting. Stallion ejaculates contain fewer viable sperm, therefore, losses and cell damage during sorting have a greater impact in horses than in bulls. Moreover, considerable stallion-to-stallion variation in semen quality, viability, sortability, and cryotolerance makes it difficult to develop a universal sorting protocol (152).

## 8 Conclusion

Flow cytometry serves as a significant tool for the multiparametric characterization of equine cells, offering substantial potential for diagnosing and monitoring immunological, inflammatory, hematopoietic, and reproductive disorders. Although equine-specific antibodies are increasingly available and research findings are promising, its application in routine equine diagnostics remains limited. This limitation is due to the absence of standardized, clinically validated protocols, reference intervals, and diagnostic thresholds. Consequently, further efforts should concentrate on validating and standardizing flow cytometric assays to enable their transition from research to clinical practice. The described method may also have valuable applications in equine andrology by allowing the simultaneous assessment of sperm viability, membrane and acrosome integrity, mitochondrial activity, oxidative stress, and chromatin status. Such multiparametric evaluation is particularly beneficial for subfertile stallions and animals of high breeding value, where accurate semen quality assessment is crucial for reproductive and economic reasons. Establishing standardized protocols and elucidating the relationship between cytometric parameters and fertility outcomes will be essential for the broader adoption of this technology in equine medicine.
