# Supplementary material for: Applications of flow cytometry in equine medicine: current clinical and research perspectives
Source: Front Vet Sci. 2026 Oct 15;13:1954604. doi: 10.3389/fvets.2026.1954604 (PMC13717631; doi:10.3389/fvets.2026.1954604)
Supplement: Supplementary file 1 [file Data_Sheet_1.pdf]

Supplementary File 1

Figure S1: Flow cytometric analysis of cells obtained from the bronchoalveolar lavage fluid (BALF) of a healthy horse. The tables compare the results of conventional cytological examination, considered the gold standard, with those obtained by flow cytometric analysis of the corresponding sample. Occasional multinucleate macrophages observed during cytological examination were likewise detected by imaging flow cytometric analysis. Using this approach, basophils cannot be reliably distinguished from other cells of the granulocytic lineage.

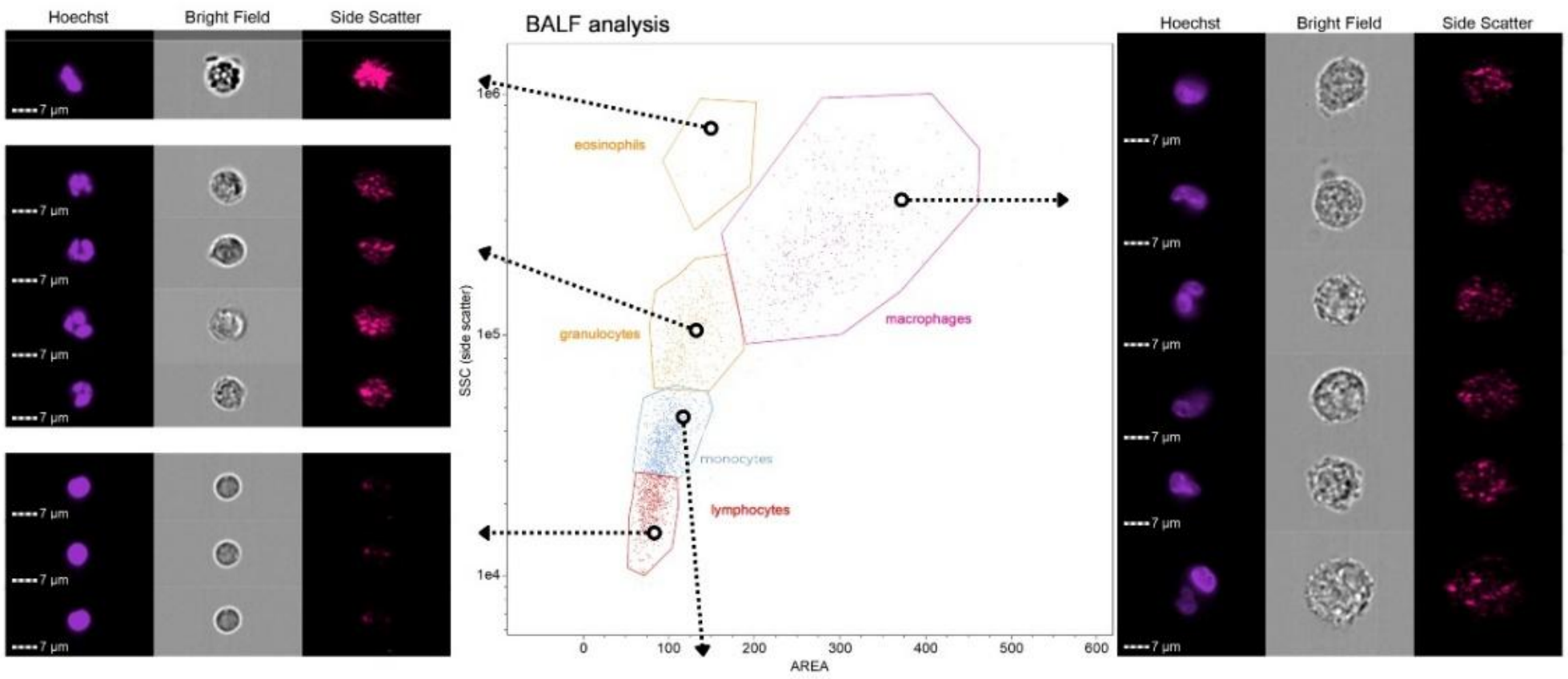

Flow Cytometric Evaluation of Cell Populations	
Neutrophils	**15,5 %**
Lymphocytes	**25,4 %**
Macrophages	17,9 %
Monocytes	36,6 %
Eosinophils	**0,2 %**

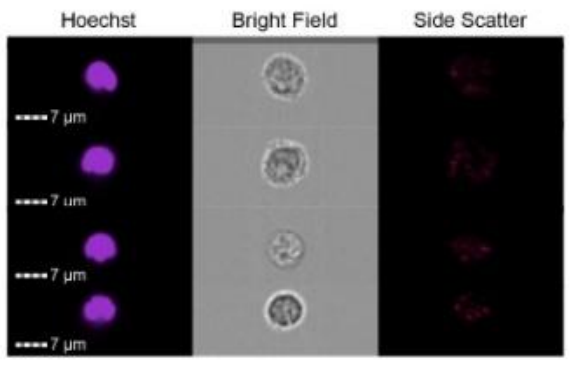

Cytological Evaluation of Cell Sediment Smears	
Neutrophils	**15,25 %**
Lymphocytes	**29,25 %**
Macrophages	51,0 %
Eosinophils	**0,25 %**
Basophils	4,25 %

Legend: Data were acquired using an imaging flow cytometer (Amnis ImageStream MkII). Nuclear staining was performed with Hoechst 33342 dye (1h in 37°C incubation). Staining served both to eliminate cellular debris from the analysis and to enable morphological evaluation of the cell nucleus. Image acquisition and analysis were conducted at 60× magnification, with the SSC laser (785 nm) operating at an intensity of 1.88 mW and 405 nm laser at intensity 10 mW. In the first step, cells showing appropriate focus (RMS_Bright Field gradient >50) were selected, followed by Hoechst-positive cells, to exclude cellular debris. In the next step, single cells were gated using the area versus aspect ratio (width-to-height ratio) plot in the Bright Field channel, defining events with an aspect ratio >0.6 as single cells. Subsequently, the cells were gated based on cell size (area) and granularity (SSC). Individual populations were delineated based on morphological features such as size, nuclear structure, and granularity; however, these are preliminary classifications and require confirmation using appropriate cellular markers.
