# Supplementary material for: Applications of flow cytometry in equine medicine: current clinical and research perspectives
Source: Front Vet Sci. 2026 Oct 15;13:1954604. doi: 10.3389/fvets.2026.1954604 (PMC13717631; doi:10.3389/fvets.2026.1954604)
Supplement: Supplementary file 2 [file Data_Sheet_2.pdf]

Supplementary File 2

Figure S2: Aspect ratio, reflecting cell shape and elongation, together with cell size (AREA), allows discrimination of epithelial cell populations in BALF samples. The sample was obtained from a horse diagnosed with severe equine asthma, characterized by marked dyspnea and a neutrophil proportion of 61% in BALF.

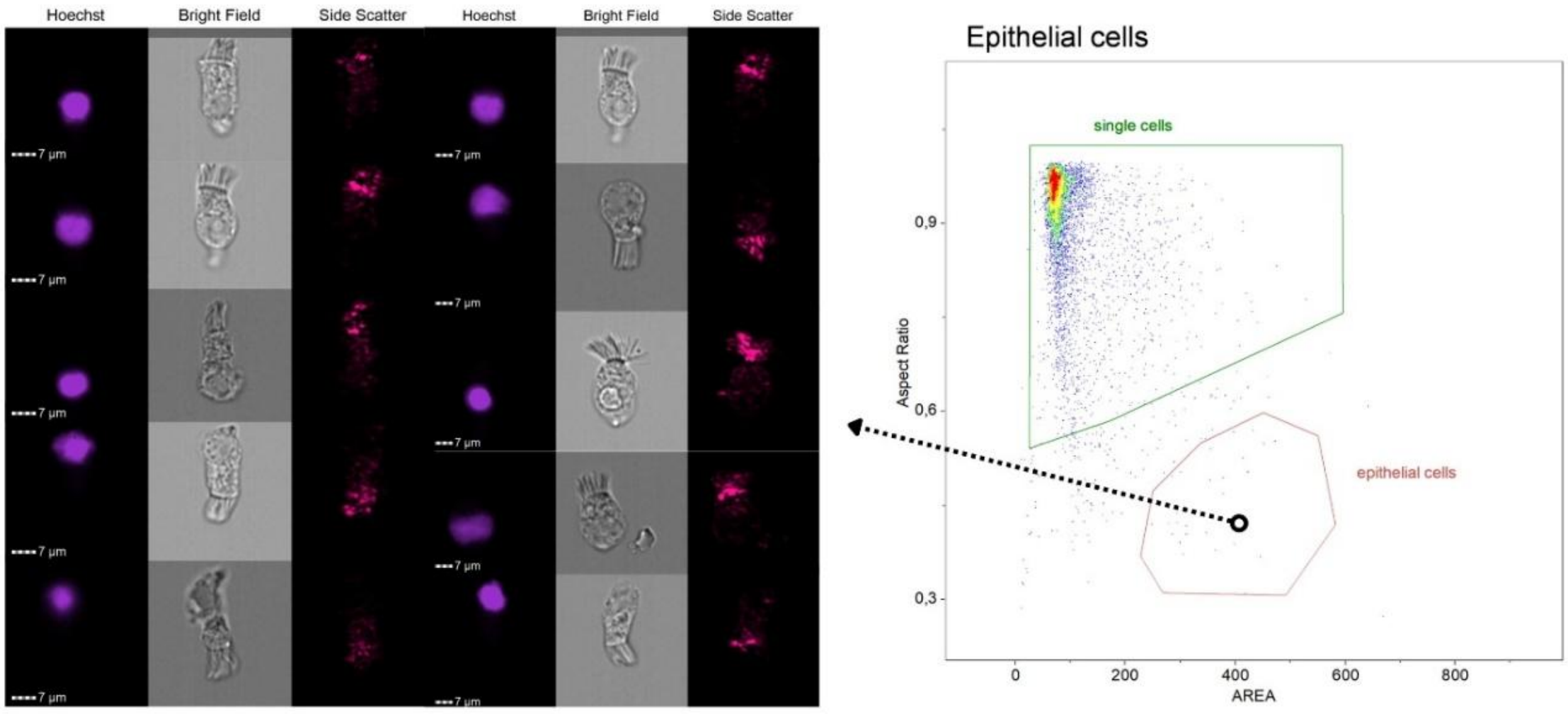

Legend: Data were acquired using an imaging flow cytometer (Amnis ImageStream MkII). Nuclear staining was performed with Hoechst 33342 dye - Staining used both to remove cellular debris from the analysis and to enable morphological assessment of the cell nucleus. Image acquisition and analysis were conducted at 60× magnification, with the SSC laser (785 nm) operating at an intensity of 1.88 mW and 405 nm laser at intensity 10 mW. In the first step, cells showing appropriate focus (RMS_Bright Field gradient >50) were selected, followed by Hoechst-positive cells, to exclude cellular debris. In the next step, single cells were gated using the area versus aspect ratio (width-to-height ratio) plot in the Bright Field channel, defining events with an aspect ratio >0.6 as single cells. During the imaging analysis of cells outside the "single cells" gate, elongated nucleated cells with a brush border were observed; these were classified as epithelial cells, however, these are preliminary classifications and require confirmation using appropriate cellular markers.
